# Southern Dental Association. Proceedings of the Annual Meeting Held in New Orleans, La. in March, 1885

**Published:** 1885-07

**Authors:** 


AMERICAN JOURNAL

OF

DENTAL SCIENCE.

VOL. XIX. THIRD SERIES—JULY 1885. No. 3.

ARTICLE I.

### SOUTHERN DENTAL ASSOCIATION.

SEVENTEENTH ANNUAL SESSION.

*Reported by Mrs. M. W. J. for Southern Dental Journal.*

Continued from Last Number.

Dr. Taft: How permanent is this effect of mercury? Is it never eradicated?

Dr. Rawls: I do not say that. Changes are taking place all the time in man, in his relations and environment. All these have their bearings on the laws of heredity.

Dr. Taft: When tissues have been affected by an agent, does the effect continue after the agent is expelled?

Dr. Rawls: The tissues can change, and do change; but an impression has been made which renders the tissues susceptible to irritants. The impresssion is made on each molecule; a change is made in the constitution of the molecules; the general impress is on the molecules, and it is transmittible; not the agent itself, as mercury, for instance, but the impress made, is permanent. It is like making a new chemical compound by the addition of another element.

Dr. Taft: The transmission of types inherit in the constitution of physical peculiarities, as in hair or eyes, is admitted by all; but that such accidents as the exhibition of medicines with evanescent effects varying with the power of the system to rally, that an impress so temporary as the effect of mercury, is transmittible, appears to me doubtful, to say the least. There are some things that are never eradicated; that are so permanent as to be ineradicable, as syphilis, for instance, which may be, and doubtless is, transmittible, but I doubt the transmission of the mercurial influence. The system may be weakened and broken down, and children consequently not robust, but the transmission of short-lived accidents appears doubtful to me. It is dangerous ground to accept such theories.

Dr. Morgan: I want to ask if all radical changes made in the system, and continued, are transmittible?

Dr. Rawls: If not, you could not live a moment. What makes you what you are? Not subtle influences that can neither be felt, seen nor handled, but the food you eat, the air you breathe; they make you what you are, and their effects upon your system and your tissues are transmittible.

Dr. Morgan: Then how is it that we who are lame all our lives have athletic sons? An eye knocked out is not transmitted. Reproductive alveolus is never in the original form. It will be largely increased in bulk and strength. I once saw a man who carried a large piece of the outer plate of his skull in his hand. New process was being formed, with healthy tissues. The periosteum is reproduced, and granulation takes place.

Dr. Robinson: Dr. Morgan has told me that the rheumatism which caused his lameness was inherited, and we do not know that he will not transmit it.

Dr. Cushing (to Dr. Rawls): What are the physical signs of these transmitted conditions?

Dr. Rawls: Pyorrhœa alveolaris presents different aspects when transmitted than when acquired. In transmis sion, the tissues are softer and less healthy; there is not the same integrity; they break down more rapidly. The lameness of Dr. Morgan not being inherited is no evidence that the conditions which made it possible were not, or that he may not transmit to future generations, though not to his son. The world did not grow in a year. The transmission is in general conditions, not in local form; in types, not the accidental impress from an abnormal element.

Dr. Walker: The fact is that the more we search for and understand causes, the better we can treat the effects or results. The observations of Dr. Rawls lead him to look upon salts and mercury as factors of causation. The deposit of calculus is evidence of misdirection of elements.

Dr. Fountain (Texas): In the Brazos bottoms, pyorrhœa is very prevalent; perhaps from malaria, perhaps from mercury. Most of my patients say they have been salivated.

Dr. Patrick: There is no doubt that the physical impress is transmitted. Proofs are very strong. A concatenation of circumstances bringing about certain results, account for those results. To illustrate: In Sedalia, Mo., a gander, in foraging for food along the railroad, got under the wheels and had his wings broken. The next spring a number of young goslings were hatched with broken wings. A photograph of the group was sent to Chas. Darwin. At his request, the gander was placed the next spring in another flock six miles away from the railroad. There was no more broken winged goslings. Circumstances and surroundings were all changed.

We will probably never know *why* one family for several generations have the two first fingers missing, even first cousins sharing the same peculiarity. There is no explaining it. It is so; that is all. The fact is established, but why or how we don't know. We find this illustrated in the teeth as in the other organs.

Dr. J. A. Robinson: I can cite one family when for four generations, to my knowledge, the grandfather, father, daughter, and her little child, have no upper lateral incisors.

Dr. Teague (S. C.): Cited the cases of two well-known families in South Carolina who have been edentulous for generations. Also another family known for three generations as inheriting a hideous deformity of double hair lip.

The discussion was closed.

Dr. J. A. Robinson, of Jackson, Michigan. then read a paper entitled "How to Increase Your Usefulness."

This paper was replete with sound advice addressed especially to the younger members of the profession, embodying the wisdom gained by the experience of a veteran in the service, "Father Jerry," as he is affectionately called by all who know him well, having passed the limit of three-score years and ten. He began by quoting the well-known saying of Daniel Webster, "There is always room at the top," regretting, however, that the most competent dentist, he who has reached the top of the ladder in professional ability and professional estimation, was appprently little appreciated by the community in which he lived, judging by his position on the financial ladder. Push, without ability, will accomplish nothing, and ability without push will not remedy this state of things. Individually, we only catch glimpses of the truth, like the gray before the dawn. It required a Franklin, then a Morse, then a Field, to perfect the telegraph. The begining of a new truth, when first seen, seems to threaten to sweep us beyond the ancient shores we thought secure. In the great inundation of the light of to-day, it is hard to distinguish what is solid ground. Our improvements grow with our affections. Being just to himself and to others, the affections of a just man are wounded by failure; he must abandon the means and measures which occasioned the failure; hence his improvement. To be successful, you must do the things that other men do, not do and to do this requires study and effort. The first means to this end is to read all the journals, that you may know what others have done, and what is left undone, for you to attempt. If the same article is published in three or four journals, read it three or four times. It would not be published so often if it was not worth it. As special fields of study, take diseased teeth and gums. This is a broad but comparatively new and uncultivated field to the average dentist. The same is true of artificial crowns, which are rarely attempted except in large cities; correcting irregularities is another important branch that is comparatively ignored by the average practitioner, while one of the most delightful branches of our art. It broadens the field, and affords relief from the ordinary routine of chair-work. It develops high art, and elevates the dentist in the eyes of community. It reveals new beauties in the human face divine, correcting deformities that would be remedied only by the sacrifice of good teeth for the substitution of artificial ones. This is a field far more practically useful than the embryonic, microscopic, and other studies upon which the student spends so much time. The cultivation of the fraternal feeling in the profession is one of the most dignified means of securing increased patronage. Let the people see that we have confidence in each other, and they will have more confidence in us. It is the rule of society everywhere. Secrecy and acquisitiveness are twin sisters; together they dwarf the mind. Hidden somewhere, like diamonds lying in the depths of the ocean, of untold value when found, are the materials capable of being so combined as to give us that ideal filling which shall truly restore the mutilated tooth to its original form and usefulness. If my premonitions are true, this will yet be found. Who among you will be the finder? One of the first qualifications for success is to have the courage of your convictions, and personal force enough to impress your patrons with the principles and truths you wish to instill; make them feel your enthusiasm as you feel it yourself. We may love the past, but we must look to the future. There is an immense realm of possibilities yet unexplored. It once seemed that Mozart and Beethoven had exhausted all the possibilities of musical sounds, but Mendelssohn and Wagner revealed new harmonies undreamed of by the old masters. Sometimes we think we love the truth when we only love our own ideas of what is true. We are here to fulfil the end for which we were created; it was not in the scheme of Providence that man should be sick, or that we should lose our teeth. The main business of the dentist is to save the natural teeth, by the best means, and with the best material within his reach. Our success will depend on our ability to make our work permanent. Artificial dentures only serve to make things bearable till we go down to the grave. A man is not fit to be a dentist unless he acts on his principle. We must deal with principles above mere mechanism. The truths of science, the laws of nature, the law of justice and right, of truth and love, must be unitized in a profession, to make it felt in the world. Cleanliness is the first law of nature; God is constantly washing all nature with His dews and rains. Man also must be obedient to this law. Filth, fermenting and putrefying, is the cause of decay. Tooth-extraction is not a part of our business. It is not a part of our mission to assist nature in working out the law of evolution by which the human race may become toothless. Edmund Burke said, "Where bad men combine, good men should combine." We should band ourselves together against the bands of charlatans. And all this we must not only believe ourselves, but we must make the world believe it. Belief lies back of conduct, as force lies back of motion. It is only what is fully believed that is acted up to. And so, the last but not the least means to increase your business will be modest, dignified advertising; direct and indirect; by card or by newspaper; by the omnipotent power of talk. By the free use of printer's ink we elevate and enlighten the world. An important matter for consideration is the lack of sufficient compensation for our labor. The world is full of equivalents; man is ever unwilling to give more than he receives.

The demand for cheap dentistry is founded in popular ignorance of the value of the teeth. Here the press is the most efficient means of spreading abroad the truth. There is less general information regarding dentistry and the teeth than on other subjects. Here we have the key to the situation. We must make the people a part of ourselves, if we expect their patronage. There are trades unions to protect the working people; there are conventions to lift the masses; there are expositions to enable the people to behold the world at a glance. We owe this to ourselves and to a long suffering humanity; to the rising generation. We owe it to the progress of the age; we owe it to a profession that has out-stripped all others in rapid advancement. Consultations in medicine are paid for, whether treatment follows or not; the lawyer has his retaining fee; the dentist is only expected to give his time for nothing—to spend more time in gratuitous examinations than he does in actual labor. Information regarding the value and the care of the teeth should be embodied in school-books, from the primary to the graduating class. Light should be shed on this subject by the daily or the weekly newspapers. The man who will thus stand by his convictions has also the right to attach his name to such information. And when we have done this, we shall have our reward.

I have followed my profession for forty-nine years, and I have always tried to do well, but I am free to confess that I have never felt the responsibility of my calling as I do to-day. Rubens said, when he was ninety years old, that he had just begun to improve, We will increase our business just in proportion as we know what to do with tooth sinners to save them, and what to say to tooth-saviors to make them faithful to their high calling.

(This is but a brief abstract of a paper which was also of practical suggestions, and information as to the latest means, methods and materials in operative dentistry.)

The Chair: This most excellent and practical paper is now open for discussion.

Dr. Taft: It is very late. I move that the discussion of this paper be postponed until the next session. Seconded and carried.

Adjourned to meet at 9 A. M,, to proceed to the grounds of the exposition, for the celebration of Dentist's Day, Thursday, April 2d.

Friday set apart for Clinics and the exhibition and explanation of Appliances.

Next regular session Friday, 7 P. M.

THIRD DAY.

Thursday, April 2d, being Dentist's Day at the World's Cotton Centenial Exposition, no regular sessions were held.

Assembling at Tulane Hall at 9 A. M., the visiting and resident dentists, with their lady friends, proceeded in a body, some two hundred and fifty strong, to the foot of Canal street, where a steamboat waited to convey them up the Mississippi river to the Exposition grounds. Their crescent-shaped badges attracted much attention, and the exclamation "What fine-looking men!" was frequently heard. The trip was a delightful one, affording a fine view of the busy city, with its warehouses and elevators lining the river's edge; its many tall steeples in the distance; the broad streets forming avenues of living green, converging to a common centre, like the sticks of an open fan, from the crescent-shaped city front. On the opposite side of the river lie the suburban villages of Gretna and Algiers, while lining the banks on either side lie at anchor vessels from across the broad ocean, and steamers from a hundred tributary streams. Reaching the Exposition landing, a halt was called on the margin of a little lake, under the shade of a gigantic live oak tree, draped with waving streamers of gray moss, where the group was photographed, facing the Exposition buildings, with the Mexican head-quarters in the rear. Thence they repaired to the Music Hall, in the main building, where front seats had been reserved for their use. Seated on the platform, and grouped around the speaker's stand, were represenatives of the Board of Management of the Exposition, and a numher of the most prominent members of the dental profession. An address of welcome on the part of the dentists of New Orleans was delivered by Dr. A. G. Fredricks, and responded to by Dr. A. O. Rawls, of Kentucky. An eloquent and appropriate address of welcome and congratulation, from Capt. S. H. Buck, Acting Director-General of the Exposition, was echoed by Col. F. C. Morehead, Commissioner-General. After brief remarks from others, and music, they repaired to the banks of Lake Rubio, to witness a special drill of the United States Life Saving Service. Thence to one of the large dining halls, where an elegant cold collation was served. The party then scattered over the grounds and buildings, to enjoy the day as best suited individual inclination. The main building, the Goverment building, the Art Gallery, the Horticultural Hall, the Mexican buildings, were all centers of attraction, the crescent badges of the S. D. A. being seen everywhere.

FOURTH DAY.

Friday, April 3d, was set apart for Clinics and the exhibition and explanation of Appliances. Mr. J. W. Selby, of the S. S. White Co., furnished the hall with all the necessary chairs, brackets, spittoons, etc. Simultaneous clinics drew together numerous animated groups.

Dr. Wm. N. Morrison illustrated the portion of the paper read by him giving his method of filling root-canals with gold wire, through a very small opening, not more than one sixteenth of an inch in diameter, in the centre of crown. The committee appointed to prepare the teeth selected those with the most crooked, curled and twisted roots that could be found in their collection, one in particular having a very remarkable double angle like a letter Z. The teeth were imbedded in blocks of plaster in such wise as to give no clue to the length, shape, or direction of the roots. The work was closely watched, and many were the predictions of the wire passing through the apical foramen on the one hand, or of failure to reach the apex on the other. The canals being first filled with gutta-percha, the slender gold wire was manipulated dexterously and rapidly, through an opening in the center of the crown scarcely larger than a pin-hole. When the work was finished, the plaster was broken away, and the teeth eagerly broken open, when lo! not a single failure was to be recorded. In every case the gold was found to reach the apex exactly, to seal it securely, and with the gutta-percha forced into all the interstices, the canal was perfectly filled. All doubts were dispelled as to Dr. Morrison's ability to do all that he claimed. Whether anyone else could do it remained an open question.

Many gathered around the chair where Dr. E. Parmly Brown, of New York, with the electric mallet and spring pluggers of his own design, built up a crown of a left superior molar which had been nearly destroyed by malpractice with the file. The pluggers used were of a new design, the portion just above the point being tempered to a degree of elasticity that obviates all risk of crumbling down the margins of enamel. The separation between the teeth for knuckling was obtained entirely by the force of the gold driving the teeth apart at the point of contact. The contour of the crown, cusps, and masticating surface was perfectly restored.

Dr. Bonwill, of Philadelphia, gave a clinic with his mechanical engine and mallet.

Order was called at 3 : 30 P. M., that those having appliances on hand might have an opportunity of explaining them. Dr. H. J. McKellops, chairman of the committee, said that there were many interesting and valuable things on exhibition, that were worthy the attention of all present; that neither trouble, time, nor expense had been spared in getting them here; that some things were patented, some were "secret preparations," some were freely given for the benefit of all, but that he hoped respectful attention would be given to each. The articles on exhibition were: A novel and very simple articulator, invented by Dr. Westmoreland, of Columbus, Mississippi; a lathe head-piece (owner and inventor not present to explain); a tooth-powder bottle stopper, with a slot in the side for convenience of placing the powder on the brush, the invention of Dr. C. E. Kells, Jr., a regulating spring for expanding the arch, invented by Dr. E. S. Talbot, Chicago. The spring is of piano wire, the tension of the spring increased by being coiled in the middle, the spring interfering much less with articulation and mastication than either jack-screws or the coffin-plate. Dr. Miles, of Charleston, exhibited a new mouth-piece for facilitating the administration of nitrous oxide gas, and dispensing with the aid of an assistant; Dr. Williams, of Kentucky, a new motor-power for engines and lathes, which is perfectly noiseless, with band which cannot slip; that gives much greater power than any other, and capable of from two to ten thousand revolutions a minute, as tested with the gauge; Dr. J. W. Smith, of Newport, Rhode Island, stiff paper disks with a rim of sand-paper for finishing cervical walls and proximal fillings; Dr. B. S. Byrnes, of Memphis, Tennessee, a new regulating appliance, or rather method, which commends itself by its extreme simplicity. It consists of merely a very thin, elastic band of 22 carat gold, hooked around the first molars, or most available posterior teeth, and bent into the interstices between the teeth. As the anterior teeth yield to the constant, gentle pressure of the elastic band, and space is gained, the band is straightened and shortened as much as necessary, and again indented to hold the teeth in the new position. This is repeated as often as necessary, until the symmetry of the arch is attained. If a gap is to be closed a gold band is hooked around the teeth to be drawn together. If special action is desired upon any one tooth, a rubber block is placed under the gold band, but no rubber rings are used, and no threads tied. Casts were exhibited showing the application of the principle, and the very remarkable results obtained in a very short time, with very little soreness of the teeth or discomfort of the patient, and very little time spent by the operator in applying the apparatus. The appliance was highly commended by Dr. McKellops.

Dr. J. A. Robinson, of Jackson. Michigan, exhibited rubber plates lined with fibrous metallic material, and also with vulcanizable gold, a material which does not corrode, or tarnish, or wear off. The injurious effects of contact of the rubber with the mucous membrane is thus avoided, while a beautiful finish is given to the plate.

Dr. Edwards, of Desmoines, Iowa, also exhibited specimens of black rubber plates made by a new method, which produces light, elastic plates, highly polished on both sides when they are taken from the flask, without the aid of any new apparatus.

Dr. J. R. Walker, of New Orleans, exhibited a modification of the Coffin plate, having the spring so adapted as to be attached to the front teeth, to draw them in while expanding the arch laterally. An interesting sketch was given by Dr. McKellops of Dr. Coffin's experience in "giving away" a good thing. At the Medical Congress in London, in 1881, when he gave this appliance to the profession, he had on exhibition a peck measure full of plates, that had done duty in more than twenty-five thousand cases. He had more than he could do with the help of two or three men, but after he had so generously demonstrated his method, everyone took it up, and his business fell away from him entirely when he no longer had the monopoly.

Dr. Bonwill exhibited his mechanical mallet and engine, explaining the difference between his and all others, the disadvantage of his electric engine and mallet being the necessity on the part of the operator for a thorough knowledge of chemistry and the laws governing electricity.

The new engine and mallet also greatly expedites the work, enabling the operator to do in forty-five to seventy minutes what would otherwise require four or five hours.

Dr. Morrison exhibited and explained some very interesting specimens of Japanese dentistry; plates with cusped teeth, carved from hard-grained wood, and stained to order, *black* for married women, as a mark of distinction and all for two or three dollars a set. Also Japanese tooth-brushes, of banyan root; the end beaten or bruised till the fibres take the semblance of a paint-brush.

Dr. Morrison also exhibited a gold crown that had been presented to the Southern Dental Association fifteen years ago, but which had been in use three years previous to that date, thus invalidating the several patents on the gold crowns now on the market.

Mr. J. W. Lambert, of the St. Louis Pharmaceutical Co., exhibited samples of Listerine and Menthated Camphor, "proprietary medicines," but not *secret preparations*, as the formula is given on the label of each bottle. Listerine, though favorably known in the medical profession for some time, has but recently been offered to the dentist, as a scientific compound possessing valuable antiseptic and disinfectant properties and specially adapted to dental and oral surgery, on account of its absolute safety, and freedom from objectionable odor and taste.

Mr. Wm. Evans, of McKesson & Robbins, New York, exhibited samples of the various preparations of cocaine, now being so largely experimented with as an obtundent of sensitive dentine, inflamed gum-tissues, exposed pulp, etc.

The Executive Committee announced the following applicants for membership:

T. J. Knapp, New Orleans.
Wm. Crenshaw, Atlanta, Ga.
R. E. Watkins, Eutaw, Ala.
A. E. Wofford, Starkville, Miss.
C. R. Rencher, Enterprise, Miss.

Who were duly elected and their names placed on the roll.

Adjourned to meet in regular session 7:30 P. M.

Friday, April 3d, 7:30 P. M.—B. H. Catching, Third Vice-President, in the chair.

Report of the Committee on Dental Literature called for.

Dr. B. H. Catching, Atlanta, Chairman, read a paper, subject, "Literature and a Prophecy" (published in June issue.)

Dr. Brown: As a guest of the Southern Dental Association, I recollect saying, on one occasion, that I was not quite full enough for utterance. Although I have been magnificently treated on the present occasion, I am not *too full* for expression. I have but a few moments to spare, however, so don't get discouraged. I shall not talk long. I am sorry to meet so few men from the North. I am sorry *for them*, for they do not know what they are losing. It has not been properly advertised. It would not be one hundred thousand men ; it would be an army of a million men that would come and capture New Orleans with the arms of friendship and love, if they knew the welcome they would meet, and what there is here for them to see. My train is probably whistling. I must bid you adieu. [applause.]

The subject of Dental Literature resumed.

Dr. E. S. Chisholm (Tuscaloosa) read a paper. (published in May issue.

DISCUSSION.

Dr. Spalding: Every profession is undoubtedly aided by its literature. In our own profession, periodicals and standard works are sufficient in quanity, but deficient in quality. Progress must be sought in that direction. Our text-books are deficient in certain branches. In materia medica and and therapeutics, almost *nil.* In other lines they have grown almost beyond our needs. Text-books are usually written by teachers. We have depended on medical men as teachers, and consequently as authors, in those branches.

They know comparatively little of their application to dental therapeutics. Very much of what we know is not in the text books; it has been learned from experience, and recorded in our journals. Medical men, as such, are not qualified to teach, or write books for, dental students. The remedy? We must bring forward men from our own ranks to teach in these departments, and to furnish the text-books we require. All the chairs in every faculty should, as a rule, be filled by dentists. It has been said that if physiology is a science, the man who can teach physiology can teach dental physiology. Practically, this is far from being a fact. Unless a man expects to become an author, he will not be thorough. There are very few thorough physiologists, even among medical men. Education in the fundamental branches is only partial. Few are qualified to teach chemistry. as applicable to our profession. Unless a man is well grounded in first principles, he is not qualified to teach. He must also understand the practical application of the principles of science to our profession. We depend on medical men to teach our students what they themselves are ignorant of. We must draw from our own ranks to supply this deficiency.

Dr. B. H. Catching: In attending associations from year to year, I see exhibited gold, amalgam, mallets, engines, etc; everything for manual use is brought before the body, but I have never seen a single volume of our literature laid upon our table: I hope our Southern Association will take steps to be furnished by publishers with copies of each and every new publication issued, to be examined by the profession at their annual meetings. I would have this made a part of the work of the Committee on Dental Literature.

Prof. J. Taft: Great improvement can yet be made, but when we look back to forty years ago, and note the improvement made in that time, in our literature, it seems almost a miracle Then we had no literature. We had but three or four standard works, and the beginning of our periodicals; but now, see our monthly supply of dental literature! I think we may conclude that some progress has been made.

Examine the pages of one of those early journals, and compare with one of to-day, and again we see the progress.

Our literature needs improvement in its literary aspect. Our permanent work—our text-books—have not kept pace with our journalism; but new men are using strong pens, and our text-books will soon equal those of other professions. Every avocation has its special literature. We must train up writers for our profession. Every man should keep a record of every step in advance. Young men should be pressed to engage in this work. If all would do this, our literature would speedily improve. Many who now write are poor writers, it is true, but we should blame no one who does his best, but encourage him to further efforts. Reference was made in the paper to lack of suitable works in certain lines. Let those who think it ought to be better strive to make it better; there is no one to hinder, the way is open. Most of the books we have are designed for those already experienced; there are no elementary books; no clear, simple books for beginners. That is the great need of to-day. It is an easy matter for those in practice to give information for those who are experienced, but it is not so easy to write for beginners. There is no elementary work on anatomy. If any one can be stimulated to prepare graded books for our profession, he will have attained a great desideratum. We need elementary books on anatomy and physiology. Would you put Gray, as a first book, in the hands of a young dental student? We should have an elementary work designed for our own students. The common school books of to-day are better than those used in our colleges. We should have a series of brief works that a student could readily master and retain. The noneclature of anatomy and pathology ought to be improved. It should be anglicized. The student needs to be familiar with two or three extra languages. The bones and muscles should have plain English names. Students must have helps to more rapid progress. What if our periodicals do contain some advertising matter? That is no disadvantage. If a man pays for 40 pages of literary matter, and gets, for nothing, 50 pages more, which tell him where to get what he most needs, is he the loser? Some men turn and look for the new advertisements first thing, and then examine the literary portion, if they have time left! Most of the journals are owned and published by the men who manufacture the goods you must have; they could not be sustained independently.

Dr. J. R. Patrick: I think Prof. Taft's position in regard to elementary works in our colleges, not tenable. There are elementary books in all our high schools, and a youth has no business in a college who has not already mastered these elementary works. Gray's Anatomy is plain and simple, and told in few words. Nothing could be plainer.

Dr. J. R. Walker: What is most needed is elementary instruction in hygiene in our primary schools. Such teachings as, when put into practice, will enable the scholars to to enter the high school, and go thence to our colleges, with sound minds insound bodies. There is much lack of primary education in hygiene. Success in after life depends on the health of the body as well as on the cultivation of the brain.

Dr. O. Salomon (New Orleans): I am surprised to hear the depreciation of American literature. No nation on earth has what America has. German and French dental literature consists almost entirely of translations and extracts from American journals. When I came to America, I thought I knew something about dentistry. I had studied in Paris, and in Berlin, but when I got to Baltimore, I found I knew nothing. As to the advertising pages, if a man has profound knowledge, his dirty coat is a matter of little consideration. The German and French journals have no advertising pages, but neither have they any reading matter worth reading.

Dr. E. S. Chisholm: Our literature is just what we make it; it is the reflex of ourselves; it is evolved from the demand. We pay for it because we feel that we need it to help us in doing our best for our patients. It is a fact well known to all who teach science that popular teachings are not correct. We must learn the first principles, in all branches, by the inductive system, gradually growing and broadening out.

Prof. J. Taft: The majority of our students, coming from the high-schools, where the elementary sciences are popularly supposed to be taught, have but the merest smattering: nothing that prepares them to take up Gray as a starting point. A student in mathematics is not given algebra or the third part arithmetic, to begin with. He must start with the elementary principle; and so it is in all branches. As geographies and arithmetics are graded, so must the sciences have graded text-books: from the prim ary schools, up through the high-schools, to the college. Prof. Ford and others have prepared question-books, and the students study them out, taking the question-books as a guide to study, and the text-books as statements of facts. We should have little primers of 20 or 30 pages, of elementary principles, in which the student should be as thoroughly grounded as in the alphabet or multiplication table. He will have from ten to twenty preliminary lectures, before undertaking laboratory work. Though foreign journals may translate or copy our record of experiences, we are indebted to them for the fundamental principles of science underlying our practicable work. Our foreign brother is warranted in speaking more freely on this point than I can do.

Dr. Spalding: I would call the attention of the Association to an illustration of the principle laid down. Gray's Anatomy is regarded as the best text-book on anatomy; the work of a most celebrated anatomical author. But turn to the pages of Dental Anatomy, and you will find a mere reproduction of that which was written by Goodsir many years ago. This fossilized statement has been handed down without alteration or comment; there is only one chapter, and that one entirely erroneous, from the modern standpoint. Must we wait for some medical man to study this subject out and write it up for us? No; we must draw upon our own resources, from our own ranks. That is the only way out.

Dr. Patrick: Gray's Anatomy is the text-book for the general practitioner of medicine; for the professional man. It does not purport to be a guide to any specialty, as of the eye, or the ear, or the throat. There are other text-books intended for men who are well-informed, but not professional. Carabelli (Vienna, 1826) gave us a valuable work in the beginning of the century, though no credit is given him. He took up the work where John Hunter left off. It is a work of world-wide celebrity, as authority of comparative anatomy. There is no lack of text-books for professional men.

Dr. A. W. Harlan (Chicago): The members of this Association are not schoolboys to be taught the rudiments. The trouble is not in the lack of text-books; it is in the lack of study of those we have. The great trouble with text-books is that they are too elementary; they are not exhaustive. There is too great a multiplication of primers. Men think they know it all, and study no further. Science is not to be taught by primers—we have health primers, science primers, biological primers, etc. They contain a mere smattering, and put forth insignificant ideas. Revised editions of the books we have, and the study of those books by the younger men, is what is needed.

Prof. Taft: The class of books that Dr. Harlan names is not what I referred to. The primers serve a good purpose. His argument would cut down the progressive, inducive system. Gray's Anatomy was not written under the histological light of to-day. It contains much that requires modifying. I agree with Dr. Catching that copies of the new publications should be brought before the association. That in itself would be a good report on dental literature. It would make a more positive impression than a written report. Would like to see such a report at every meeting.

Dr. B. H. Teague (Aiken, S. C.): I think it only right that this Association should take public recognition of the work of Mrs. M. W. J.; a work that has not only a national reputation, but a world-wide fame. I move that we tender the thanks of this Association to the author of the book entitled, "Letters from a Mother to a Mother."

Prof. J. Taft moved an amendment adding that the Association recommend the book to all practicing dentists for circulation.

Dr. C. W. Spalding: Having had an opportunity of examining a copy of the recent revised edition, I would say that it does not do justice to the author. The workmanship does not correspond with the contents. The work itself is the very best of its class; has no equal, in my judgment, for placing in the hands of mothers.

The motion, as amended, passed unanimously, and was ordered so recorded.

Prof. Taft moved that the author be requested to prepare a revised edition free from the errors and faults referred to. Had examined the book, and was prepared to endorse what Dr. Spalding said of its value. It was intended for distribution among patients, but every practicioner ought to read it. Moved that the author be requested to prepare an edition free from error.

Dr. C. W. Spalding: Though agreeing with the sentiment, would doubt the expediency of the measure, as an edition of 5,000 were just from the press, and the resolution would imply censure of the publishers, as the faults were faults of workmanship and typographical errors, etc.

"Mrs. M. W. J." was introduced to the members of the Association, and in a few words expressed her gratification at the reception her mork had met, and the kind words spoken, explaining that the text had been very carefully revised for the new edition, and that the typographical and other errors were entirely due to the haste in which the new edition had been issued, in order to have it out in time for examination by the Association; that haste, however, having defeated its own object.

The motion of Dr. Taft was withdrawn.

The subject of Dental Literature was passed.

Dr. H. J. McKellops desired to call attention to the subject of the Code of Ethics—that he should probably tread on somebody's toes, but was only sorry for their corns! He wished to call attention to the matter of giving certificates—a subject to which he had been giving some attention. He knew that itinerants were in the habit of cutting out these certificates and pasting up whole columns, to which they call the attention of patrons. Many of our own members have their name in print, attached to recommendations of articles of which it is very doubtful if they know the constituents. Look at the list of certificates to Holmes' Sure Cure! Do they know what this is? I propose to buy a bottle and present it to our distinguished chemist, Dr. Harlan, for analysis. I have fought this practice for years. If you want to get your name honorably before the public, buy a quantity of the books of the lady just mentioned, put your name on them, and distribute them far and wide. It will be better for yourselves, and good for your patients.

Report of the Committee on Clinics called for.

Dr. McKellops moved that the subject be passed, as the time was very short, and there was much business that must be transacted—the election of officers; selection of time and place of meeting, etc.

Carried.

Proceeded to ballot for time and place of meeting.

Nashville was selected, the time being fixed for the fourth Tuesday in May, 1886.

Drs. E. Telle, New Orleans, and M. S. Read, Corsicana, Texas, were elected members and enrolled.

The election of officers was now had, resulting in the election of

Dr. C. W. Wardlaw, for President.

Dr. B. H. Catching, of Atlanta, Ga., First Vice-President.

Dr. J. Rollo Knapp, of New Orleans, La., Second Vice-President.

Dr. E. D. Hammer, of Galveston, Texas, Third Vice-President.

Dr. E. S. Chisholm, Tuscaloosa, Ala., Corresponding Secretary.

Dr. R. A. Holliday, of Atlanta, Ga., was re-elected Recording Secretary.

Dr. H. A. Lowrance, of Athens, Ga., was re-elected Treasurer.

Dr. W. H. Morgan, of Nashville, Tenn.; Dr. G. F. S. Wright, of Columbia, S. C., and Dr. W. H. Richards, of Knoxville, Tenn., were elected as the Executive Committee.

The newly elected officers were then inducted into office.

Dr. Jas. S. Knapp conducted the newly elected President to the chair. In a few well-chosen words, Dr. Wardlaw returned thanks for the honor conferred, begging the cordial support and co-operation of the members to make the next meeting equal the present in interest and benefit; proposing to make his administration a working one. He hoped that all members of committees would willingly fulfil their duty, or give early intimation of inability to do so—let us come up as a host, supporting and helping each other, making this a great society for good.

"Mrs. M. W. J." was elected an honorary member of the Association; the Secretary requested to give formal notification of the same.

The following resolutions were introduced by Dr. J. R. Walker, of New Orleans, and unanimously adopted:

*Resolved*, That the Southern Dental Association desires to extend a vote of thanks, in token of its profound appreciation of the very generous action of the Board of Management, the Trustees and Faculty, of Tulane University, in affording to us and to the National Board of Dental Examiners the free use of their hall and committee rooms, day and night, during the full term ef our meetings. Also to Mr. J. W. Selby, for his liberality in furnishing the hall with the chairs and other appliances necessary for clinics. Also for his general assistance in receiving and entertaining the members of the Association. Also to the press of the city of New Orleans for their enterprising daily reports of the proceedings.

*Resolved*, That the thanks of the Association be tendered the retiring president, Dr. A. O. Rawls, for the able and judicious manner in which he has performed the onerous duties devolving upon him during his term of office.

Dr. Clifton, of Waco, Texas, moved that the thanks of the Association be tendered the dentists of New Orleans for the pleasant entertainment at the World's Exposition. Carried.

Dr. Walker moved that the Secretary and Treasurer be included. Carried.

Adjourned to meet in Nashville, Tenn., on the 4th Tuesday in May, 1886.

